# A Participatory Health Promotion Mobile App Addressing Alcohol Use Problems (The Daybreak Program): Protocol for a Randomized Controlled Trial

**DOI:** 10.2196/resprot.9982

**Published:** 2018-05-31

**Authors:** Robert J Tait, Jessica J L Kirkman, Michael P Schaub

**Affiliations:** ^1^ National Drug Research Institute Faculty of Health Sciences Curtin University Perth Australia; ^2^ Hello Sunday Morning Sydney Australia; ^3^ Swiss Research Institute for Public Health and Addiction University of Zurich Zurich Switzerland

**Keywords:** alcohol drinking, internet, evaluation studies, social marketing, health promotion

## Abstract

**Background:**

At-risk patterns of alcohol use are prevalent in many countries with significant costs to individuals, families, and society. Screening and brief interventions, including with Web delivery, are effective but with limited translation into practice to date. Previous observational studies of the Hello Sunday Morning approach have found that their unique Web-based participatory health communication method has resulted in a reduction of at-risk alcohol use between baseline and 3 months. The Hello Sunday Morning blog program asks participants to publicly set a personal goal to stop drinking or reduce their consumption for a set period of time, and to record their reflections and progress on blogs and social networks. *Daybreak* is Hello Sunday Morning’s evidence-based behavior change program, which is designed to support people looking to change their relationship with alcohol.

**Objective:**

This study aims to systematically evaluate different versions of Hello Sunday Morning’s *Daybreak* program (with and without coaching support) in reducing at-risk alcohol use.

**Methods:**

We will use a between groups randomized control design. New participants enrolling in the *Daybreak* program will be eligible to be randomized to receive either (1) the *Daybreak* program, including peer support plus behavioral experiments (these encourage and guide participants in developing new skills in the areas of mindfulness, connectedness, resilience, situational strategies, and health), or (2) the *Daybreak* program, including the same peer support plus behavioral experiments, but with online coaching support. We will recruit 467 people per group to detect an effect size of *f*=0.10. To be eligible, participants must be resident in Australia, aged ≥18 years, score ≥8 on the alcohol use disorders identification test (AUDIT), and not report prior treatment for cardiovascular disease.

**Results:**

The primary outcome measure will be reduction in the AUDIT-Consumption (AUDIT-C) scores. Secondary outcomes include mental health (Kessler’s K-10), days out of role (Kessler), alcohol consumed (measured with a 7-day drinking diary in standard 10 g drinks), and alcohol-related harms (CORE alcohol and drug survey). We will collect data at baseline and 1, 3, and 6 months and analyze them with random effects models, given the correlated data structure.

**Conclusions:**

A randomized trial is required to provide robust evidence of the impact of the online coaching component of the *Daybreak* program, including over an extended period.

**Trial Registration:**

Australian New Zealand Clinical Trials Registry ACTRN12618000010291; https://www.anzctr.org.au/Trial/Registration/TrialReview.aspx?id=373110 (Archived by WebCite at http://www.webcitation.org/6zKRmp0aC)

**Registered Report Identifier:**

RR1-10.2196/9982

## Introduction

Globally, 5.9% of deaths are attributed to alcohol consumption, and 5.1% of the global burden of disease and injury is attributed to its use [1]. In Australia, alcohol consumption is estimated to cause 3.2% of the total burden of disease. Including harms to nonusers, alcohol consumption contributes about 188,000 disability-adjusted life years, causes 5550 deaths, and costs approximately AU $30 billion/year [2,3]. Australian guidelines provide recommendations for the general adult population specifying that, to reduce the risk of alcohol-related harm over the lifespan, average consumption should be 2 or fewer drinks per day. To reduce the risk of injury from a single occasion of drinking, 4 or fewer drinks are recommended. Further, more stringent recommendations are made for specific groups (eg, pregnant women, those with a family history of alcohol disorders) where the risk of alcohol-related harms is greater [4].

Considered against the 2 main guidelines, in 2016, about 17% of adults were at-risk from their average alcohol consumption, about 26% from their single-occasion use [5], and 37% from either single occasion or average use [6]. Notably, most alcohol-related harm arises from those who consume in an at-risk manner rather than those with an alcohol disorder, with a much greater number of at-risk drinkers than those with disorders [7]. Furthermore, less than 17% of those with an alcohol disorder ever receive treatment for their condition [8], and among those people with less severe alcohol problems, most are unlikely to seek help for their drinking [9].

One approach to addressing alcohol use among those with less severe problems is opportunistic screening and brief intervention (SBI), with the evidence base for the effectiveness of SBI being substantial [10-13]. Under the rubric of SBI, programs can range from a simple screening question combined with a minimal amount of feedback, eg, normative data on alcohol use, through to the use of standardized screening instruments and more intensive interventions that can include components of motivational interviewing and cognitive behavioral therapy. Typically, SBI can be incorporated into a single treatment session in a few minutes but can be several sessions lasting a few hours [14]. However, some, but not all, studies suggest that SBI may be more effective with males than females, and there is an absence of evidence for their effectiveness for those with more severe alcohol use problems [10,11,15]: these people are typically referred for more intensive treatment [16]. Overall, SBI does not appear to have been widely adopted into primary care, and questions remain about its practical implementation, especially within the context of the Australian drinking culture [17,18].

Therefore, alternative approaches to complement existing programs are required. Web-based SBI alcohol interventions have been developed, evaluated, and found to be effective in both college student samples and the general public, excluding students [19,20], with typical effects in the small to medium range (eg, effect size *g=*0.20-0.44) [19,21]. Web-based interventions in their briefest form (eg, single session) typically rely on normative feedback on the self-reported quantity of alcohol consumed, whereas more complex interventions may involve multiple sessions and draw on a range of psychological approaches including motivational enhancement, cognitive behavior therapy, and behavioral control [19,21], similar to the components used in traditional SBI. Nevertheless, Web- or mobile-based SBI has potential advantages over traditional formats, including extended reach, anonymity, timely accessibility, treatment fidelity, the potential to model behavior, and access without the involvement of professionals [21,22]. The evidence to date for the effectiveness of mobile phone–based alcohol interventions is inconclusive, with outcomes ranging from significant improvements on some alcohol measures to significant declines on other measures [23,24].

The benefits of including additional resources to support internet-based interventions are currently unclear. A meta-analysis of internet interventions for depression found a large effect size (*g*=0.78) [25], where all except one of the studies included therapist support. In contrast, a meta-analysis of self-guided internet interventions for depression reported a small effect size (*d*=0.28) [26]. However, a comparison of alcohol interventions found no significant difference between guided (effect size *g*=0.23) versus unguided (effect size *g*=0.20) interventions, although the authors note that this may be due to the small number of guided studies [19]. Nevertheless, benefits have been shown in improving alcohol outcomes (and other health behaviors) from using multiple modes of delivery such as text messages, drawing on theoretical models, and incorporating multiple behavioral change techniques [27,28].

Observational data show that a person’s social network can either increase or decrease their alcohol use [29]. Social networks now include those formed on the internet. These sites range from simple bulletin boards through to virtual lives, but typically allow users to post blogs (text) and images that stimulate discussion and the formation of social groups [30]. The effects of these networks on behavioral change have been evaluated with inconsistent effects found with respect to diet and exercise [31], but to date, the effectiveness of social networks in reducing alcohol consumption has not been determined [30,31], but it is currently being trialed in youth with depression and who engage in binge drinking [32]. In contrast, the persuasive effects of Web-based social networks have been extensively harnessed by alcohol brands to engage with alcohol consumers, with some brands having attracted millions of followers [33,34]. This trial will evaluate the effectiveness of the online coaching component of Hello Sunday Morning’s *Daybreak* program and associated support as a means of reducing alcohol consumption.

Hello Sunday Morning is an Australian social media health promotion movement that asks participants to publicly set a personal goal to stop drinking or reduce their consumption for a set period of time (typically 3, 6, or 12 months [35]), and to record their reflections and progress on blogs and social networks [35,36]. The platform was created in 2010, with the aim of motivating members and holding them accountable by asking them to set a public goal. Further development included gamification features, which were structured games that facilitated participation and engagement with the internet community. Participants in the legacy Hello Sunday Morning platform were recruited by social marketing tactics, leveraging the idea of a movement toward a better drinking culture through social media, word of mouth, and a television commercial. A large portion of members were also recruited through Google AdWords. This platform is no longer actively recruiting participants, and as such individuals are now directed toward the *Daybreak* program.

Initial research conducted on the platform explored the blog content and qualitative reports of change. Text analytics of the blog posts on the platform show that participants typically begin with descriptions of their drinking practices, which over time change to posts reflecting their efforts to change their behavior [37]. Research on the Australian users has found that 84% of participant’s report completing the program time they signed-up to without a slip-up, defined as drinking alcohol before finishing. These participants reported reduced alcohol use and desire to drink alcohol for fun or to relieve tension, and improved mental health [38]. Participants have also reported feeling more positive about themselves, improved productivity and engagement in new activities, as well as new and/or improved relationships and financial savings [39].

Australian users of Hello Sunday Morning are more likely to be female (61%) than male, with most aged less than 50 years (89%). Few (5%) are classified as low-risk alcohol users as assessed by the alcohol use disorders identification test [40] (AUDIT) (score=0-7), with 42% classified in the hazardous or harmful range (AUDIT 8-19) and 53% classified as probably dependent (AUDIT≥20) [35]. Limited follow-up data (n=49) on a subsample of Victorian Hello Sunday Morning participants (n=345) found that the prevalence of probably dependent drinkers fell from 45% at baseline to 7% at 1 month and 24% at 3 months [41].

The aim of the study was to evaluate the effectiveness of the online coaching component of the *Daybreak* program in reducing alcohol consumption via a randomized controlled trial. The *Daybreak* program is a smartphone app developed by Hello Sunday Morning, based on the original Web-based program. *Daybreak* consists of the same social-networking peer-support forum as the Hello Sunday Morning program but also includes clinically based activities known as behavioral experiments and chat-based coaching. The primary hypothesis is that those randomized to receive *Daybreak* including peer-support, behavioral experiments (see *Content of the interventions* for details) plus online coaching, will show a greater reduction in alcohol-related problems that those randomized to just access the *Daybreak* program including peer-support and behavioral experiments (without online coaching). Consistent with the literature, the secondary hypotheses are that greater improvements will be shown for those with lower AUDIT scores (8-19), than those in the higher score category (≥20), and finally, greater improvements are expected for male participants [15,42].

## Methods

### Design

**Figure 1:**
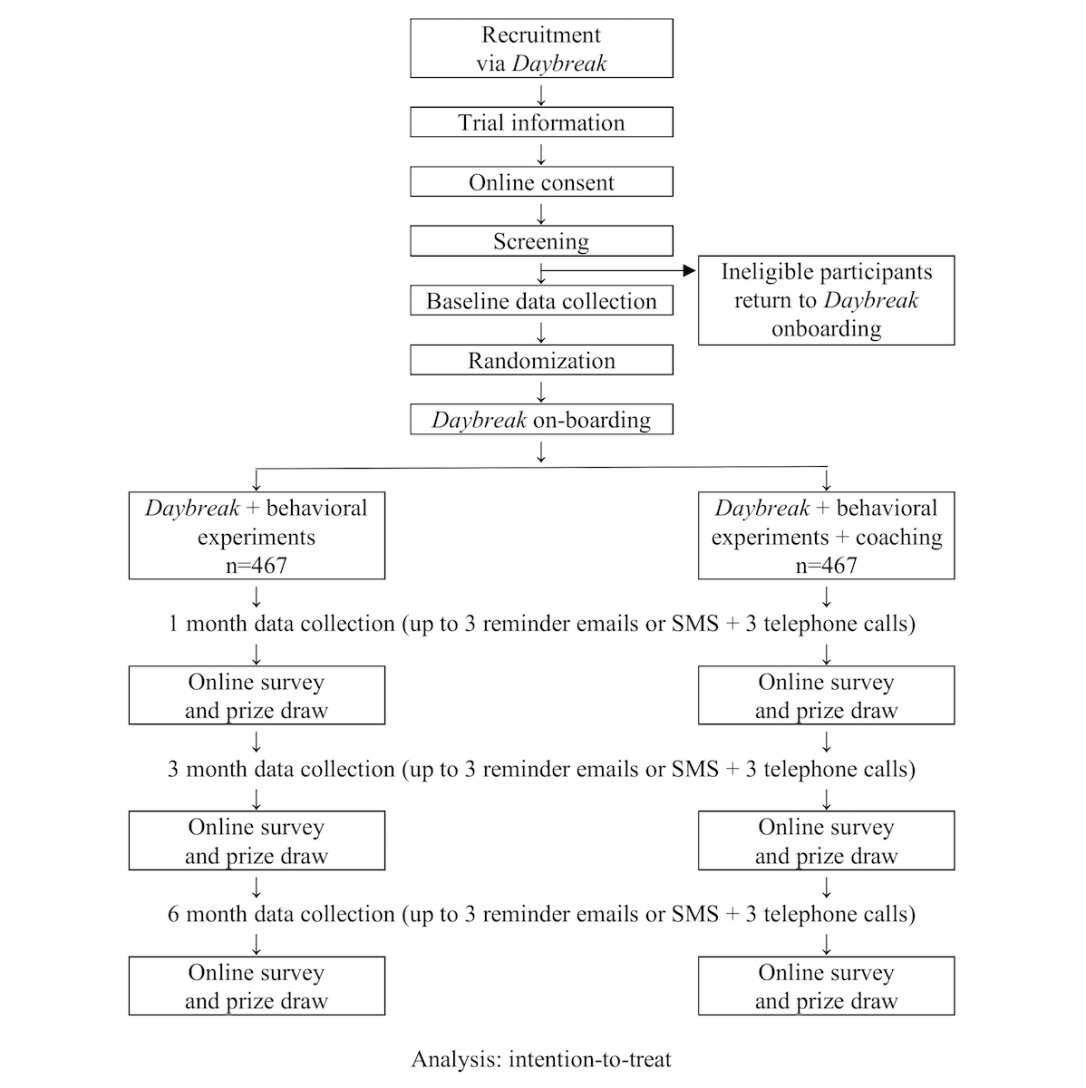
Flowchart of participants through the trial.

We will use a randomized control design to compare (1) the *Daybreak* program including peer support, behavioral experiments plus online coaching or (2) *Daybreak* program including peer support and behavioral experiments. (The content of the interventions are described below.) Outcome data will be collected at 1, 3, and 6 months post enrollment in the study with all groups receiving the same schedule of assessments (Figure 1). Australian ethical guidelines preclude the use of placebo (no treatment) control groups where a risk has been identified and there are existing treatments/interventions [43].

### Participants and Randomization

All new registrants to *Daybreak* will receive information about the study: it is anticipated that no additional advertising will be required. To be eligible for the study, participants must be aged 18 years or older, score 8 or more on the [44] AUDIT, and be resident in Australia. Participants also require a valid email address and access to the internet. We will exclude those who have ever received treatment for cardiovascular disease, due to the risks for this latter group [45]. In addition, the screening questions include the P4 suicidality screening survey [46]. Those scoring above minimal risk will still be eligible for the study but will receive details of a national, 24-hour crises support line (lifeline).

Regardless of the group to which they are randomized, those scoring 20 or more on the AUDIT (probable dependence) will be eligible for inclusion in the study but will also be advised to seek in-person guidance from their general practitioner or other clinician before reducing their alcohol consumption, to minimize the risk of serious complications from alcohol withdrawal. Given the large sample, simple fully automated randomization will be implemented. Participants will be blind to their group allocation—all will receive an active treatment. Participants access the platform by logging in with their email address and password.

### Procedure

Enrollment and intervention procedures will be self-guided. Individuals who register to start the *Daybreak* program generate a username and password, and they will then be asked if they are interested in participating in the project. For those affirming, they will then receive the full study description and be asked for active consent (clicking on a button). They will then be screened for eligibility by completing a Web-based survey, before being randomized. Those that are screened as ineligible are returned to the *Daybreak* on-boarding procedure (Multimedia Appendix 1) and notified of their ineligibility, and they are provided with relevant details of the crisis support services. Participants have unlimited access to their respective areas, and those randomized to *Daybreak* plus coaching can access a health coach (available from 7 AM to 7 PM local time Monday-Friday).

At 1, 3, and 6 months, participants will be emailed and texted a link to the follow-up survey. If they do not complete the survey, a repeat text message will be sent 1 week later. Following this, a research assistant (blind to group allocation) will call the participants to remind them to complete the survey on a maximum of 3 occasions and provide the link again via email or text message if requested during the call. At each wave, participants will be invited to enter their details for a prize draw to win an iPad 2. Participants do not have to complete the survey to enter the draw, but the link to the prize draw will be at the end of the surveys. The study received institutional ethics approval (2017-0855) and has been registered with the Australian and New Zealand Clinical Trials Registry trial number ACTRN12618000010291.

Recruitment will be conducted over 10 months from February to November 2018 or until the desired sample size is obtained. New registrants either directly download the *Daybreak* app from the App Store, or reach the Hello Sunday Morning website (where they are directed to download it) by searching Google, or by clicking from other websites such as healthdirect.gov or other health service directories, or by clicking through from the Hello Sunday Morning email newsletter.

### Sample Size

We are unaware of any prior studies using social networks for alcohol reduction, but for other behaviors, the reported changes are small but not significant [31].

The effect sizes for Web-based SBI alcohol interventions are in the range *d*=0.3-0.4 at 6 months [19-21]. On the basis of a small estimated effect size of *f=* 0.10 (equivalent an effect size of *d*=0.2) at 6 months, with a correlation between repeated measures of *r*=0.5, we will require 60 people per group for the main analysis based on power of 0.80 and alpha set to *P*<.05. Given the interest in the effectiveness of interventions by gender and for those with probable dependence (assessed via the AUDIT), the objective will be to recruit approximately 60 people in the smallest cell(s). From demographic data [35] with about 50% of the *Daybreak* population likely to be in the probable dependence AUDIT category and 40% being male, to obtain about 60 people in this cell will require 300 people per study arm (600 in total). To allow for attrition, the target will be to recruit 467 per arm (N=934), with 35% lost to follow-up.

### Outcome Measures

All measures will be assessed at each time point except the AUDIT (baseline only) and adverse events [47] (baseline and the final time point; see Table 1). The primary outcome will be change in AUDIT-consumption (AUDIT-C) scores. The AUDIT was developed by the World Health Organization and validated in a range of countries, including Australia [44]. It has a range of 0-40: a threshold of 8 or more will be used as a screening measure to identify eligible participants at baseline. With follow-up at 1, 3, and 6 months, the change in the first 3 questions (known as the AUDIT-C) will be the primary outcome and used to assess change in alcohol use (as some of the other AUDIT questions use a reference period of the last 12 months). The AUDIT-C has also been shown to predict clinical outcomes at 1 year [48]. The secondary outcomes will be changes in:

1. Alcohol consumption assessed with a 7-day drinking-diary [49,50].
2. Kessler’s K-10 is a brief screening instrument for psychological distress and also a treatment outcome measure [51]. Scores range from 10 to 50, with scores of 20-24 interpreted as showing a mild distress, 25-29 a moderate distress, and 30-50 showing a severe mental health distress.
3. Kessler’s Days out of role [52] will be used to quantify days either completely or partially out of role in the previous 30 days and the number of these due to alcohol use. Those with alcohol dependence have significantly more days completely or partially out of role than those without a disorder. Similarly, those with comorbid substance use and anxiety disorders or substance, anxiety, and affective disorders have increased days out of role [52].
4. Health resource use will be assessed with a checklist of medical and other health professionals consulted in the last 8 weeks and adapted from an existing checklist [53] to include alcohol or other drug treatment services and alcohol pharmacotherapy.
5. The global rating item from the Pittsburgh Sleep Quality Index will be used to categories sleep quality on a 4-point scale (very bad-very good) in the previous month [54].
6. The 3-item Godin Leisure-Time Exercise questionnaire will be used to quantify the frequency of spending 15 minutes or more engaged in each of 3 levels of exercise intensity (strenuous, moderate, and light) in the last 7 days [55].
7. Quality of life will be assessed with the 8-item EUROHIS-QoL that has been validated in Australia [56] and is recommended for use in alcohol and other drug treatment services [57].
8. Adverse events associated with alcohol use will be assessed at baseline and 6 months with the checklist from the Core alcohol and drug survey [47]. It was designed specifically for college populations, so 2 items specially referencing this setting were modified (ie, “missed a class” changed to “missed a class or work” and “been in trouble with police, resident hall, or other college authorities” changed to “been in trouble with police or other authorities”). Given the focus on alcohol, we deleted the reference to drug use from the lead in statement and from 1 item (“Thought I might have a drinking or other drug problem”). Among college students in the United States in the previous 12 months, more than one-third reported driving under the influence and 20%-30% had been in an argument or fight.

### Content of the Interventions

The *Daybreak* program is designed for people looking to change their relationship with alcohol. It allows participants to set a goal, reflect on their mood, and engage with peer support. *Daybreak* helps people to change their relationship with alcohol in 4 ways.

The first one is weekly check-ins. *Daybreak* supports members in a self-reflection process to discover their inner drivers. With the help of Australia’s leading experts in motivational interviewing, *Daybreak* has created a set of self-report questionnaires (Multimedia Appendix 2) that are designed to help people uncover their intrinsic motivators for change.

The second one is peer support. *Daybreak* connects members to the *Daybreak* Community, a channel to immediate empathy, problem solving, and accountability. Currently, 45% of shares in *Daybreak* receive 5 or more comments within an hour. The program supports a vibrant community with members who care for each other, help each other navigate tough times, and keep each other in check.

**Figure 2:**
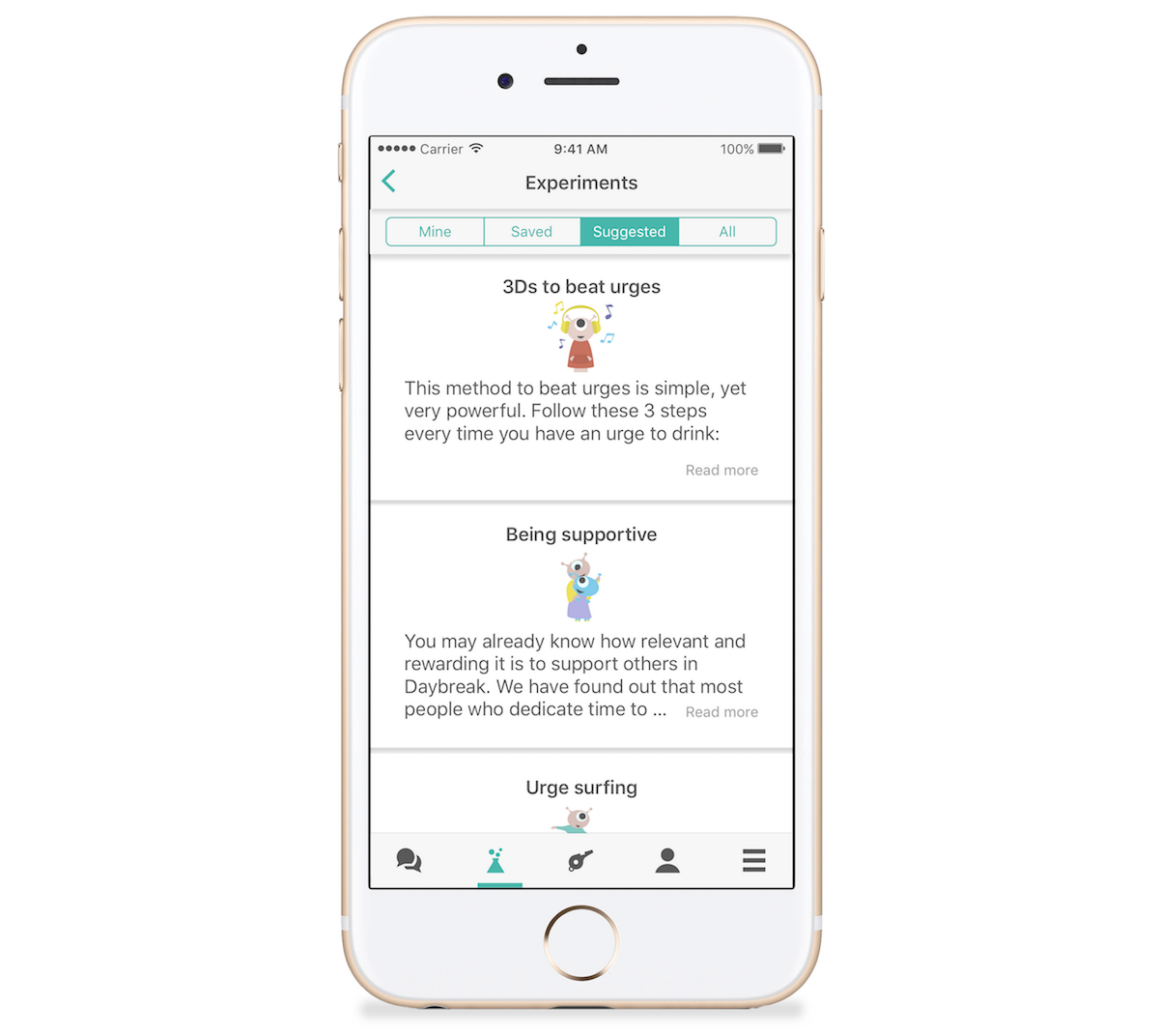
Example of Daybreak behavioral experiments.

The third one is behavioral experiments. *Daybreak* encourages members to take experiments and reflect on their learnings. Designed by the Hello Sunday Morning Clinical Team, these experiments draw on techniques from cognitive behavioral therapy, motivational interviewing, and acceptance and commitment therapy. There are a range of experiments across 5 areas including mindfulness, connectedness, resilience, situational strategies, and health. Mindfulness experiments help members learn how to be in-the-moment and recognize when a drinking trigger has been activated. The connectedness experiments help them to connect with others in a meaningful and healthy way without alcohol. The resilience experiments teach them how to bounce back from negative experiences and lapses. The situational experiments provide strategies for a range of common trigger situations. Finally, the health experiments assist members in establishing good fitness routines, good eating and sleeping habits, and general health strategies to help with regulating mood (Figure 2).

The fourth one is health coaches. All health coaches meet the low-intensity mental health guidelines set by the Australian Government Department of Health [58]. Specifically, this includes a minimum certificate IV in a mental health or community services discipline. The clinical team of health coaches at Hello Sunday Morning all meet this minimum criterion, as well as having specific alcohol and other drug training and experience. Some coaches are registered General and Clinical Psychologists.

Health coaches are required to undertake in-house training, which includes modules on health coaching and how it differs from therapy, ethical considerations when providing Web-based services, alcohol facts and management strategies, forming connections during online support, motivational interviewing, case studies and ethical dilemmas, coaching procedures, risk management, as well as platform-specific training modules. They undergo a period of supervision on initial interactions with participants and then periodically receive feedback from a senior health coach. Health coaches also participate in fortnightly peer supervision for case discussion and receive individual supervision with an Australian Health Practitioner Regulation Agency–approved supervisor who is a clinical psychologist, when needed.

Health coaches partner with members to help them set and reach goals for satisfying and healthy lives. The coaching service is conducted via real-time chat-based messaging on a secure platform. Health coaches again use cognitive behavioral therapy, motivational interviewing, and acceptance and commitment therapy techniques. Coaches tailor support and the approaches used to suit the member’s needs.

### Analysis

We will evaluate the primary and secondary outcome using an intention-to-treat approach with the effect of the intervention on each measure being assessed via a time-by-group interaction. Due to the correlated data arising from the repeated measures, we will employ a multilevel mixed effects regression model with a random intercept term. This will control for clustering of variance within individuals over the repeated measures. For continuous data, we will use an unstructured correlation matrix with a normal distribution and identity link. For other types of data (eg, count, categorical), multinomial, Poisson, or negative binomial distribution will be used with their appropriate link functions. A sensitivity analysis will include use of specialist alcohol services or alcohol pharmacotherapy as a covariate.

### Participant Safety

There are a number of mechanisms in place for forum monitoring (eg, blog posts and comments). The first is an automated system that alerts the clinical team when a member writes a post including a trigger word and receives more than 30 comments. Trigger words are predetermined words that may indicate a member is at risk (eg, kill, suicide, hurt, end it, no meaning, hopeless, cannot cope, die). Second, the forum is monitored by support staff and the clinical team, who will intervene on a thread if needed and contact the member privately via email. Third, members are able to report posts to *Daybreak* administration if they are concerned about a member or their post.

The existing *Daybreak* Risk Management Protocol will be used for all participants in the *Daybreak* research trial who report serious mental health concerns, suicidality, self-harm, family violence, or risk to children. Although there are procedures in place for each concern, they typically include working collaboratively with the member to keep them safe, deescalating the situation and implementing a safety plan, as well as encouraging them to contact suitable referrals (eg, specific or face-to-face services) provided by health coaches.

**Table 1:** Data collection schedule.

Measure	Baseline	1 month	3 months	6 months
Demographics	✓			
Drinking diary	✓	✓	✓	✓
AUDIT^a^	✓			
AUDIT-C^b^	✓	✓	✓	✓
K-10^c^	✓	✓	✓	✓
Days out of role	✓	✓	✓	✓
Health resources	✓	✓	✓	✓
Sleep quality	✓	✓	✓	✓
Exercise	✓	✓	✓	✓
Quality of life	✓	✓	✓	✓
Adverse events	✓			✓

^a^AUDIT: alcohol use disorders identification test.

^b^AUDIT-C: alcohol use disorders identification test-consumption (items 1-3).

^c^K-10: Kessler 10.

The Hello Sunday Morning site and intervention are not designed to provide support for emergency situations, such as severe withdrawal symptoms (seizure or delirium tremens) or severe psychological distress. In a general hospital sample, 0.65% incurred either a seizure or delirium tremens [59], but in an alcohol detoxification inpatient sample, 5.6% had delirium tremens and 7.4% had seizures [60]. Therefore, anyone scoring 20 or more on the AUDIT will be advised to seek medical guidance before reducing their alcohol use. We will also recommend that anyone who has ever incurred a seizure or delirium tremens seek advice. In addition, we will provide details of national hotlines for psychological problems.

## Results

Enrollment of participants started in February 2018, with recruitment projected to be completed in November 2018, with the 6 month follow-up completed by the end of May 2019. Data analysis has yet to start.

## Discussion

To the best of our knowledge, this will be the first formal evaluation of the effectiveness of a smartphone-based peer support platform with coaching in reducing alcohol consumption and associated harms. It will assess critical, clinically relevant outcomes, including changes in alcohol consumption and improvements in mental health and quality of life. The minimal use of incentives in the project design will mean that the findings will be generalizable to real-world settings, with the long period of follow-up important in establishing the persistence of any behavioral changes.

Currently, there is no definitive evidence to support the effectiveness of standard brief interventions among those with more severe alcohol disorders, with equivocal findings for both reductions in alcohol use and improving treatment seeking [15]. Nearly half of Hello Sunday Morning participants score in the probable dependence range on the AUDIT [35], so the outcomes for this subgroup of participants is of particular importance in addressing the gap in the provision of effective alcohol consumption interventions.

The study has been powered to detect effects within critical subgroups, namely, those with people with potential alcohol dependence and by gender. Therefore, in addition to reporting statistical significance results for the main analyses, which may represent trivial changes given the overall size of the sample, we will report effect sizes and discuss effects in terms of their clinical significance, eg, changes in categorical levels on the K-10 and AUDIT.

With respect to the continuing provision of the service, *Daybreak* is a service of the organization Hello Sunday Morning, which is funded through a range of commercial and corporate partnerships, grants, and government contracts. These will allow ongoing dissemination of the service including hosting the program and funding its health coaches. If successful, outcomes of the study will be used to advocate for further funding and wider dissemination via other sites, such as general practitioners and hospital emergency departments.

The study raises some ethical issues, particularly with respect to the age of participants and the adverse outcomes that can occur in unsupported withdrawal from alcohol use. Although we will ask participants to confirm that they are aged 18 years or older, and thus able to provide informed consent, the noninvasive nature of the intervention reduces the concerns about underage participants entering the trial. To mitigate against the potential for unsupported severe withdrawal symptoms, we will advise participants considered to be at higher risk to seek professional guidance before reducing or ceasing consumption.

The study relies on self-reported data, which is common for Web-based alcohol interventions [19]. Other than the identification of acute intoxication, objective quantification of alcohol use even over a limited period (such as 7 days) is difficult. None of the standard clinical markers (eg, gamma glutamyltransferase) perform adequately as screening tests for this task, particularly with women and at intermediate levels of alcohol use, or provide a quantification of the amount of alcohol consumed [61]. Therefore, we believe that there is no other method of determining the extent of alcohol use that is more reliable than self-report, without implementing far more demanding procedures, such as twice daily breath testing, as used in some judicial programs [62].

## Appendix

Multimedia Appendix 1 On boarding questionnaire and feedback at sign up.

Multimedia Appendix 2 Weekly review questionnaire.

## Abbreviations

AUDIT: alcohol use disorders identification test
AUDIT-C: AUDIT-consumption
K-10: Kessler 10
SBI: screening and brief intervention
