# Supplementary material for: A Participatory Health Promotion Mobile App Addressing Alcohol Use Problems (The Daybreak Program): Protocol for a Randomized Controlled Trial
Source: JMIR Res Protoc. 2018 May 31;7(5):e148. doi: 10.2196/resprot.9982 (PMC6002672; doi:10.2196/resprot.9982)
Supplement: Multimedia Appendix 1 [file resprot_v7i5e148_app1.pdf]

Demographics

1) I am ...

- a woman | a man | other gender

2) I live...

- by myself | with my partner | with the kid(s) | with housemate(s) | with my parent(s)

3) Are you of Aboriginal and/or Torres Strait Islander origin?

- Yes | No

Goal Setting

4) A year from now, what would you like your relationship with alcohol to look like?

[Input text]

Short term goal

5) Now let's break it down. What will be your first short-term goal? Something that you can realistically achieve in 3-6 months. [Input text]

We'll be checking in weekly to review your journey and you'll be able to change this anytime from your profile.

6) What's your focus to begin with?

- Quit drinking
- Reduce my drinking
- Maintain my current achievements

7) In the past week, how often did you feel the urge for a drink?

- Never
- Occasionally
- Quite often
- Daily or almost daily
- All the time

Stage of change

8) Have you already tried cutting back on your drinking?

1. Not yet
2. Yes but it hasn't worked
3. Yes and it worked for some time
4. Yes and I'm still not drinking or I'm drinking less

   Feedback participants receive based on their answer:
   1. Well, we're glad you're here! (Daybreak has a team of qualified coaches for this purpose)
   2-3 That's okay, it takes practice. We'll help you find what works best for you so we can achieve your goals.
   4 That's great! Please share what works for you in Daybreak later on. The Daybreak community could benefit from your experiences. [Skip alcohol questions and receive the following feedback for maintenance]

Maintenance option feedback:

To keep up with a healthy lifestyle remember:

- You have a goal to focus on: [input user's goal]
- Your support to the community can keep you motivated and it is incredibly valuable.
- Now we are ready to join the community

Let's go! [Opens community feed]

Now we'll guide you through some self-reflection to help you get started.

9) At the moment, how ready do you feel to change your relationship with alcohol?

1. Couldn't be more ready
2. I'm ready
3. I'm considering it, but I have mixed thoughts
4. I'm not ready yet but I am going to do some research

   Feedback participants receive based on their answer:
   [If ready: 1,2] Good start! You're on the right path when you're ready to change.
   [If not ready: 3,4] Many people are initially uncertain about changing the way they drink. Part of getting ready to change is hearing other people's stories and understanding your own experience. We suggest you read through the Daybreak feed especially when you're feeling doubtful for some more information about what helps people successfully change.

Here is a map of the stages of change to help you understand how the process usually works.

10) In what kind of situation do you usually drink?

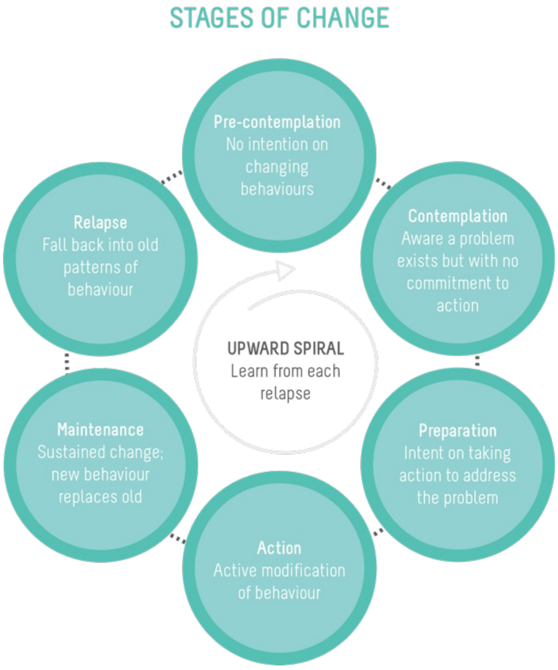

- After work
- In the evening
- During the day
- On weekends at home

- On weekends going out
- After putting the kids to bed [Filter out this option for people with no kids]
- Other (Input text)

Now we'll use AUDIT-C, a screening test by The World Health Organization to assess your drinking habits.

11) How often do you have a drink containing alcohol?

- Never [skip question 12, proceed to question 14]
- Monthly or less
- 2-4 times a month
- 2-3 times a week
- 4 or more times a week

12) How many drinks containing alcohol do you have on a typical day?

- 1 or 2
- 3 or 4
- 5 or 6
- 7 to 9
- 10 or more

13) How often do you have six or more drinks on one occasion?

- Never
- Less than monthly
- Monthly
- Weekly
- Daily or almost daily

From your answers we can see that your current relationship with alcohol is:

Feedback participants receive based on their answer
A. [If user is male and AUDIT-C score is ≥4 or if user is female and AUDIT-C score ≥3]
at a risky level regarding your health and possible harm. You can improve your health, mind, and relationships by gradually reducing your alcohol consumption. We strongly suggest that you visit your doctor before making changes to the way you drink.

B. [If user is male and AUDIT-C score is 1 to 3 or if user is female and AUDIT-C score 1 to 2]
of low risk. You can still improve your health, emotions, and relationships by drinking less. Your current situation can help making changes surprisingly easy.

C. [If AUDIT-C answer to question 11= 0]
Great! You probably want to maintain an alcohol free or a limited alcohol consumption, we are here to help.

Discrepancy Questions

14) What are the effects of alcohol in your life that you would like to change?
[eg. hangovers...]

15) What would your life without those effects of alcohol be like?
[eg. I would have active mornings]

Remember:

- You have a goal to focus on: [user’s goal]
- You have downloaded Daybreak so you are ready to change.
- And last but not least, you are not alone. You have a supportive community waiting for you here with Daybreak!

Now we are ready to kick off this journey to make better choices!

Let’s go! [Opens community feed]
