# Supplementary material for: A Participatory Health Promotion Mobile App Addressing Alcohol Use Problems (The Daybreak Program): Protocol for a Randomized Controlled Trial
Source: JMIR Res Protoc. 2018 May 31;7(5):e148. doi: 10.2196/resprot.9982 (PMC6002672; doi:10.2196/resprot.9982)
Supplement: Multimedia Appendix 2 [file resprot_v7i5e148_app2.pdf]

1. How would you rate your effort towards your goal: [input participants personalised goal]

- I didn't need to try; it was easy. [Feedback: This is great! Remember to keep checking in even when things are going well. This is a great way to support others who are finding it more difficult.]
- I tried really hard. [Feedback: Nice work! Think about what you found most helpful and share your tips with the community]
- I put in a decent effort. [Feedback: Can you identify the thoughts or activities that motivated you? Talk to our Health Coaches* if you want to work on ways to improve your efforts.]
- Could do better. [Feedback: That's okay! Think about what you might want to try next and where to find motivation. Remember that a Health Coach* is always here to help.]
- I didn't try at all. [Feedback: There's still time today! Think about what you might want to try next and where to find motivation. Remember that a Health Coach* is always here to help.]

*** Only those with access to health coaches see references to health coaches**

2. In the past week, how often did you feel the urge for a drink?

- Never
- Occasionally
- Quite often
- Daily or almost daily
- All the time

3. How intense were your urges on average? [Response option is a slider bar with minimum label "Not Intense" and maximum label "Very intense"]

4. How many times did you drink as a result of those urges? [Response option is a slider bar with minimum label "Never" and maximum label "All the time"]

Feedback participants receive based on their answer:
[If Never] Well done! You are surfing your urges.
[If any other response] Don't worry, this is normal. Let's have a look at the rest of your week.

5. What do you want to try next week? We'll suggest an experiment specially for you during the week.

- A social experiment
- A health experiment
- A mindfulness experiment
- A positive psychology experiment
- I'll think about it during the week

6. Remember to reward yourself for your efforts, and even small achievements. What will be your reward today? [Text input with placeholder text that says: For example: by going to the movies, remember to avoid rewards where alcohol might be a temptation.]
